# Frequency and risk factors for the birth of small-for-gestational-age newborns in a public maternity hospital

**DOI:** 10.1590/S1679-45082016AO3684

**Published:** 2016

**Authors:** Marina Parca Cavelagna Teixeira, Tatiana Peloso Reis Queiroga, Maria dos Anjos Mesquita

**Affiliations:** 1 Hospital Municipal Maternidade Escola de Vila Nova Cachoeirinha “Dr. Mario de Moraes Altenfelder Silva”, São Paulo, SP, Brazil

**Keywords:** Infant, newborn, Birth weight, Risk factors, Fetal growth retardation, Morbidity

## Abstract

**Objective:**

To determine the frequency and risk factors of small-for-gestational-age newborns in a high-risk maternity.

**Methods:**

This is an observational, cross-sectional, and case-control study, conducted in a public tertiary care maternity hospital. Data from 998 newborns and their mothers were collected through interviews and review of medical records and prenatal care cards. Some placentas underwent histopathological analysis. The variables of small-for-gestational-age and non-small-for-gestational-age newborns and of their mothers were statistically compared by means of Student's *t* test, Fisher's exact test, and odds ratio. The significance level used was 0.050.

**Results:**

There was a 17.9% frequency of small-for-gestational-age newborns. The statistically significant factors associated with the birth of these babies were female sex (p=0.012); positive history of another small-for-gestational-age child (p=0.006); inadequate prenatal care (p=0.019); smoking (p=0.003); hypertensive disorders of pregnancy (p=0.007); placental bleeding (p=0.009) and infarction (p=0.001).

**Conclusion:**

In the population studied, the frequency of small-for-gestational-age newborns was high and associated with sex, inappropriate prenatal care, presence of maternal diseases and addictions, and placental abnormalities.

## INTRODUCTION

According to Anderson et al.,^(1)^ the interest for small-for-gestational-age (SGA) newborns began when it was observed that babies classified as SGA, appropriate-for-gestational-age (AGA), or large-for-gestational-age (LGA) had specific morbidity and mortality for each birth weight (BW) class, according to the gestational-age (GA).

The adequacy of fetal growth for GA came in 1967, with the introduction of the intrauterine growth curve developed by Battaglia et al.,^(2)^ describing the relation between BW and GA. From then on, the identification of SGA, AGA, and LGA infants at birth became possible.^(2–4)^

Other classification criteria were developed afterwards, based on specific population studies, such as the methods of Alexander et al.,^(5)^ Williams et al.,^(6)^ among others.

Over 70% of SGA occur due to constitutional factors, such as female sex, ethnicity, parity or maternal body mass index (BMI). In these situations, the SGA newborns are not at risk of perinatal morbidity and mortality.^(4)^

Approximately 30% of SGA neonates are subject to early and late disorders, after inadequate weight gain for GA.^(1,4)^

A few years ago, the main concern was to reduce complications and improve survival of SGA infants in the immediate neonatal period. Currently, more emphasis is being placed on long-term complications, including short stature, obesity, cardiovascular diseases, hypertension, polycystic ovary syndrome, and type 2 *diabetes mellitus*.^(7)^

The consequences of being SGA encouraged the authors of this study to determine the frequency and risk factors of SGA in a public maternity hospital for high-risk pregnancy in São Paulo. The data obtained in this study may contribute to the development and implementation of measures that result in reducing the incidence of SGA infants.

## OBJECTIVE

To determine the frequency and risk factors of small-for-gestational-age newborns in a public maternity for high-risk pregnancy.

## METHODS

This was an observational, cross-sectional, case-control study of live births, which was conducted at a tertiary public maternity hospital in the city of São Paulo. The study was carried out from March 16 to June 10, 2014.

Included were all neonates born between zero o'clock on Sunday and 11:59 p.m. on Wednesday, for operational reasons of the researchers. Exclusion criteria were infants who died at birth, children of mothers who refused to participate, and babies born between zero o'clock on Thursday and 11:59 p.m. on Saturday.

Then, medical data on the newborns and mothers were collected from medical and prenatal care records, which were confirmed by the mothers during a structured interview, conducted by the researchers within the first 24 hours after delivery.

The variables studied in the mothers included age; number of consultations in the prenatal care and GA upon initiating prenatal care; schooling years; number of pregnancies; parity; use of legal (tobacco and ethyl alcohol) and illegal (marijuana, crack, cocaine) drugs; previous SGA child; weight gain during pregnancy; BMI; family income in minimum wages; number of people living in the same house; serology (toxoplasmosis, rubella, cytomegalovirus, hepatitis B and C, human immunodeficiency virus (HIV) and Venereal Disease Research Laboratory (VDRL)); clinical complications during pregnancy; hypertensive disorders of pregnancy (HDP); GDM; urinary tract infection (UTI); pre-gestation comorbidities; chronic hypertension (CH); *diabetes mellitus,* among others. An adequate prenatal care included at least six appointments, one in the first quarter, two in the second quarter, and three in the third quarter.^(8)^

The variables studied in the newborns were sex (male, female, unspecified); BW; GA; classification of newborns according to GA (preterm with GA <37 weeks, term with 37 weeks ≤GA ≤41 6/7 weeks, and post-term with GA ≥42 weeks);^(9)^ adequacy of newborns based on BW and GA, according to Alexander et al.,^(5)^ because this is the classification method adopted as the standard at the *Hospital Municipal Maternidade Escola de Vila Nova Cachoeirinha “Dr. Mario de Moraes Altenfelder Silva”* (SGA – BW *versus* GA < 10^th^ percentile, AGA – 10^th^ percentile ≤ BW *versus* GA ≤ 90^th^ percentile, and LGA – BW *versus* GA > 90^th^ percentile);^(2,5,9)^ classification of newborns according to BW (extremely low weight with BW <1,000g; very low weight with BW <1,500g; and low weight with BW <2,500g).^(1)^

The placentas were weighed and sent for histopathological analysis. The presence of alterations that could interfere with the BW of the neonates was assessed.

The comparisons of the newborn groups (SGA and not SGA) in the demographic and clinical variables were performed by the parametric Student's *t* test for quantitative variables, and by Fisher's exact test for qualitative variables.

The risk of being SGA for each variable was estimated by odds ratio (OR). In order to define the risk factors among the demographic and clinical variables, we applied the multivariate logistic regression method.

The family income was estimated as the median divided by the number of people living in the same house.

The placental changes were analyzed in a subsample of newborns whose highest incidence was SGA. This bias was controlled by a weighting system.

It was considered as significant the probabilities associated with test values equal to 0.050 or lower.

The study started after approval by the Research Ethics Committee of the *Hospital Municipal Maternidade Escola de Vila Nova Cachoeirinha “Dr. Mario de Moraes Altenfelder Silva”*, under the resolution number 528.568, CAAE: 25893114.1.0000.5454, and after having an Informed Consent signed by the mothers, which was designed for the specific purposes of this study.

## RESULTS

During the study period, there were 1,856 hospital live births and 9 out-of-hospital live births, totaling 1,865 newborns. A total of 998 newborns met the inclusion criteria, and their characteristics are shown in table 1. The remaining 867 infants were excluded because they were born between zero o'clock on Thursday and 11:59 p.m. on Saturday.

**Table 1:** Characteristics of the newborns

Variables		n (%)
Sex		
	Female	514 (51.5)
	Male	484 (48.5)
	Unspecified	0 (0.0)
Birth weight (g)		
	<1,000	11 (1.1)
	1,000–1,499	11 (1.1)
	1,500–2,499	91 (9.1)
	≥2,500	885 (88.7)
Gestational age		
	Preterm	106 (10.6)
	Term	882 (88.4)
	Post-term	10 (1.0)
Classification		
	SGA	179 (17.9)
	Non-SGA	819 (82.1)
	Total	998 (100.0)

SGA: small-for-gestational age; g: grams.

We carried out the histopathological study of 340 (34%) placentas, of which 127 (37.4%) were from SGA infants. Single or multiple changes, when present, were more frequent in SGA infants (Table 2).

**Table 2:** Placental histopathological changes

		SGA n (%)	Non-SGA n (%)	Total n (%)
Placental changes				
	Yes	91 (71.7)	135 (63.4)	226 (66.5)
	No	36 (28.3)	78 (36.6)	114 (33.5)
	Total	127 (100.0)	213 (100.0)	340 (100.0)
Type of change				
	Intervillous fibrin deposition	42 (46.2)	66 (48.9)	108 (47.8)
	Infarction	29 (31.9)	34 (25.2)	63 (27.9)
	Retroplacental haematoma	4 (4.4)	3 (2.2)	7 (3.1)
	Calcification/microcalcifications	33 (36.3)	53 (39.3)	86 (38.1)
	Acute chorioamnionitis	13 (14.3)	20 (14.8)	33 (14.6)
	Necrosis	1 (1.1)	3 (2.2)	4 (1.8)
	Vascular congestion	5 (5.5)	11 (8.1)	16 (7.1)
	Fibrinoid degeneration	1 (1.1)	0 (0.0)	1 (0.4)
	Intervillous hemorrhage	5 (5.5)	2 (1.5)	7 (3.1)
	Chorioangioma	1 (1.1)	0 (0)	1 (0.4)
	Total	91 (147.4)	135 (142.1)	226 (144.2)

SGA: small-for-gestational age.

The family income was less than one minimum wage *per capita* in 806 (80.8%) mothers. A total of 17 (1.7%) women did not received prenatal care, and in 804 (81.9%), the prenatal care was adequate, according to the policy of the Brazilian Ministry of Health.^(8)^ Other maternal data are described in table 3.

**Table 3:** Maternal characteristics

Characteristic		General n (%)	SGA n (%)	Non-SGA n (%)
Maternal age (years)				
	<16	27 (2.7)	3 (1.7)	24 (2.9)
	16–34	857 (85.9)	150 (83.8)	707 (86.3)
	>34	114 (11.4)	26 (14.5)	88 (10.7)
	Total	998 (100.0)	179 (100.0)	819 (100.0)
Schooling level (years)				
	≤3	15 (1.5)	4 (2.2)	11 (1.3)
	4–7	227 (22.7)	54 (30.2)	173 (21.1)
	8–11	661 (66.2)	105 (58.7)	556 (67.9)
	>11	95 (9.5)	16 (8.9)	79 (9.6)
	Total	998 (100.0)	179 (100.0)	819 (100.0)
Parity				
	Nulliparous	428 (42.9)	76 (42.5)	352 (43.0)
	1–3 births	532 (53.3)	98 (54.7)	434 (53.0)
	>3 births	38 (3.8)	5 (2.8)	33 (4.0)
	Total	998 (100.0)	179 (100.0)	819 (100.0)
Prenatal care				
	Yes	981 (98.3)	171 (95.5)	810 (98.9)
	No	17 (1.7)	8 (4.5)	9 (1.1)
	Total	998 (100.0)	179 (100.0)	819 (100.0)
Previous SGA newborn				
	Yes	145 (26.7)	33 (34.7)	112 (24.9)
	No	399 (73.3)	62 (65.3)	337 (75.1)
	Total	544 (100.0)	95 (100.0)	449 (100.0)

SGA: small-for-gestational age.

At some point during pregnancy, 115 (15.7%) mothers smoked tobacco cigarettes. Of these, 29 (19.1%) stopped using them during pregnancy. During pregnancy, 49 (4.9%) women ingested alcohol; 29 (2.9%) used illicit drugs; 5 (17.2%) used *crack*, 11 (37.9%) used cocaine, and 15 (51.7%) used marijuana.

Due to the lack of data, maternal weight gain and BMI of early pregnancy were not obtained.

Among the mothers who underwent serology tests, none tested positive for rubella and cytomegalovirus during pregnancy. A total of 3 (0.3%) mothers had positive serology for toxoplasmosis, 3 (0.3%) for HIV, and 3 (0.3%) for hepatitis C, all of them with non-SGA children. VDRL was positive in 17 (1.7%) mothers, and 6 of them had SGA children and 11 had non-SGA children. The positivity of hepatitis B virus occurred in six (0.6%) participants whose children were non-SGA.

The maternal clinical changes, isolated or associated, are shown in table 4.

**Table 4:** Maternal clinical changes in the current pregnancy

Clinical conditions		SGA n (%)	Non-SGA n (%)	Total n (%)
PIH				
	No	152 (84.9)	746 (91.1)	898 (90.0)
	Yes	27 (15.1)	73 (8.9)	100 (10.0)
GDM				
	No	169 (94.4)	754 (92.1)	923 (92.5)
	Yes	10 (5.8)	65 (7.9)	75 (7.5)
UTI				
	No	115 (64.2)	525 (64.1)	640 (64.1)
	Yes	64 (35.8)	294 (35.9)	358 (35.9)
CH				
	No	173 (96.6)	803 (98.0)	976 (97.8)
	Yes	6 (3.4)	16 (2.0)	22 (2.2)
DM				
	No	179 (100.0)	813 (99.3)	992 (99.4)
	Yes	0 (0.0)	6 (0.7)	6 (0.6)
Other				
	No	151 (84.4)	725 (88.5)	876 (87.8)
	Yes	28 (15.6)	94 (1.5)	122 (12.2)

SGA: small-for-gestational age; PIH: pregnancy-induced hypertension; GDM: gestational *diabetes mellitus*; UTI: urinary tract infection; CH: chronic hypertension; DM: *diabetes mellitus*.

Using the Fisher's exact test and p significance level, there was an association between SGA infants and female sex (p=0.002), multiple gestation pregnancy (p=0.001), maternal schooling level of four to seven years (p=0.040), previous SGA children (p=0.050), and no prenatal care (p=0.005).

Using risk analysis by OR, female sex was 1.69-fold more likely to be SGA than male sex (p=0.002); multiple pregnancy increased 3.48-fold (p<0.001) the risk of SGA: maternal schooling had no significant increased risk (p=0.169); the presence of previous SGA children increased the risk 1.6 fold (p=0.050), and the absence of prenatal care increased 4.21 fold (p=0.002) the risk of SGA.

Using univariate analysis, there was no significant relation between SGA newborn and adequate or inadequate prenatal care (p=0.124), maternal age (p=0.114), number of previous births (p=0.759), and *per capita* income (p=0.675).

The Fisher's exact test and p significance level indicated that smoking, drinking and illicit drug use during pregnancy were related to the birth of SGA neonates (p<0.001).

The Student's *t* test showed that the amount of cigarettes was not significantly associated with the birth of SGA babies (p=0.575).

There was a favorable trend for the birth of SGA and the gestational period of smoking cessation by the Fisher's exact test. The later the discontinuation, the greater the probability the child be SGA (p=0.023).

In risk analysis, smoking increased 2.58 times the probability of a SGA (p<0.001) newborn, whereas alcohol consumption increased it 3.12 times (p<0.001), and the use of illicit drugs increased it 5.26 times (p<0.001). However, the only drug that was statistically significant was *crack* (p=0.042).

Applying the Fisher's exact test and p significance level, the gestational clinical conditions associated with the birth of SGA were syphilis (p=0.032), and HDP (p=0.019). However, this did not occur with GDM (p=0.348), UTI (p=1.000), DM (p=1.000), and CH (p=0.260).

There was a risk tendency to a SGA newborn in pregnant women with syphilis (2.61-fold more likely; p=0.054) and HDP (1.82-fold more likely; p=0.013), using risk analysis by OR.

Placentas less than 700 g were associated with the birth of SGA babies (p<0.001) by the Fisher's exact test and p significance level. In risk analysis, placentas weighing less than 500g had 7.81-fold higher risk of SGA neonate (p<0.001) when compared to placentas weighing more than 700g.

A study weighted by the Fisher's exact test showed a statistically significant association between the birth of SGA babies and the histopathological changes in the placenta (p=0.047). Placental infarction (p=0.029) and intervillous hemorrhage (p=0.009) were significant. In risk analysis, placentas with any histopathological changes had 1.45-fold higher risk of SGA baby (p=0.039). In placental infarction, the risk increased 1.56-fold (p=0.027), and in intervillous hemorrhage the risk increased 4.13 times (p=0.003).

Multiple regression analysis identified the increased risk factors for SGA as inadequate prenatal care, 1.6-fold higher risk (p=0.019); previous SGA child, 4.33-fold (p=0.006); smoking, 1.9-fold (p=0.003); PIH, 1.54-fold (p=0.007); female sex, 1.54-fold (p=0.012); intervillous hemorraghe, 4.28-fold (p=0.009), and placental infarction, 2.01-fold (p=0.001). Alcohol consumption tended to be a risk factor, with borderline p value (p=0.074).

## DISCUSSION

The importance of classifying newborns, associating their weight to GA, lies in the fact that SGA newborns have specific morbidity and mortality rates.^(1)^

Establishing the SGA frequency was the objective of several nationwide studies, but a precise comparison of them is limited due to the use of different classification methods by the hospitals. In addition, the data available in Brazil, from the Liveborn Information System (SINASC), do not allow for the identification of births that are below the 10^th^ percentile for each week of pregnancy.^(10)^

A study conducted by Almeida et al.,^(11)^ in the city of Santo André (State of São Paulo), showed a frequency of 4.3% of SAG neonates, classified by the curve of Lubchenco et al.,^(12)^ Ragonesi et al.^(13)^ In the city of São Paulo, in 1993, a total of 4,9% of SAG newborns were found by the same curve. Zambonato et al.,^(14)^ from the city of Pelotas (State of Rio Grande do Sul), in 1996, showed a frequency of 13.1% of SAG neonates, classified by the intrauterine growth curve of Williams et al.^(6)^ A study performed by Costa et al.,^(15)^ in the city of Cotia (SP), in 2009, reported an incidence of 3.5% SAG newborns classified by the curve of Lubchenco et al.^(12)^

In this study, the frequency of SGA infants was 17.9%. This rate may have been higher than in previous studies due to the fact that this study was conducted in a reference maternity hospital for high-risk pregnant women, presenting with various diseases and lower socio-economic conditions, which are factors associated with increased birth of SGA newborns.^(4)^

Some authors determined the association of low maternal age and the birth of SGA babies.^(16)^ Almeida et al.,^(11)^ showed no association between increased incidence of SGA and young pregnant women. These authors, as well as Odibo et al.,^(17)^ associated age of the mother greater than 35 years with an increased frequency of SGA. In contrast, in this study, maternal age was not statistically associated with the birth of SGA infants (p=0.114), both by multivariate analysis and univariate analysis.

It is accepted that nulliparity increases the risk of SGA infants when compared to multiparity,^(18)^ as shown by Thompson et al.,^(19)^ in three hospitals in Auckland, New Zealand. In this study, no association was observed between the number of previous deliveries and an increase in the frequency of SGA.

Several studies, such as those carried out by Bakewell et al.,^(20)^ found a relation between previous SGA child and greater probability that the next child will also be SGA. The same was found in this study, in which a previous SGA child increased the probability of the current baby also being SGA.

The rate of SGA infants is considerably higher among disadvantaged economic groups and those with lower schooling level.^(11)^

Almeida et al.,^(11)^ identified the low level of maternal education as a risk factor for SGA. Mothers with incomplete elementary school education showed 70% greater chance of having a SGA child than those with higher levels of education. Among pregnant women with university level, no SGA newborn was found.

Similarly, this study observed a statistically significant association between low schooling level and the risk of birth of SGA neonates, most frequently in mothers with four to seven years of schooling.

In this study, univariate risk analysis showed that mothers with family income below one minimum wage were 1.78 times more likely to have SGA children.

Adequate prenatal care is important to prevent the birth of SGA infants. Kilsztajn et al.,^(21)^ by the analysis of data collected regarding prenatal care, provided the diagnosis and treatment of numerous complications during pregnancy and reduced the risk factors for the birth of SGA babies.

In this study, by univariate analysis, mothers who received prenatal care had lower incidence of SGA children compared with those who did not, regardless of the number of appointments. In multivariate analysis, inadequate prenatal care, according to the Brazilian Ministry of Health criteria,^(8)^ has been shown as a risk factor for the birth of SGA infants.

Maternal exposure to legal and illegal substances is a major risk factor for changes in fetal growth and development.^(18)^ Illicit drug use is associated with SGA neonates. The use of illicit drugs is usually accompanied by the use of alcohol and tobacco and is strongly related to socioeconomic factors.^(18,22)^

A study conducted by Mesquita et al.,^(22)^ showed that the greater the amount of alcohol consumed by pregnant women during the first and second trimesters of pregnancy, the lower the BW, the head circumference and the height of newborns.

Smoking is dose-dependent for the birth of SGA. It has been discussed in the literature if the lesions caused by smoking may be reversible if the mother quits smoking at some point during pregnancy.^(18)^

The Screening for Pregnancy Endpoints (SCOPE)^(23)^ reported that women who stopped smoking at about 15 weeks of gestation had an SGA newborn rate similar to the group of non-smokers.

Fergusson et al.^(24)^ discussed the use of marijuana and its effects during pregnancy. Newborns of women who made use of this substance showed lower weight than the newborns of those who did not use it. After adjustment for confounding factors, the association had borderline significance for the birth of SGA infants.

A review study conducted by Cembranelli et al.,^(25)^ found that usually newborns of women who abused cocaine were born prematurely, with low birth weight and height, and head circumference smaller than normal.

Mello^(26)^ showed a direct association between the use of *crack* during pregnancy and the birth of SGA babies, in a maternity hospital, in the city of São Paulo.

By analyzing the data of maternal exposure to legal and illegal substances in this study, smoking, alcohol consumption, and illicit drug use are strongly related to an increased probability of SGA, respectively in 2.58; 3.12 and 5.16 times.

As for smoking, in this study, the amount of cigarettes did not affect SGA incidence. However, there was a trend toward increased incidence of SGA – the later the discontinuation of the drug, the greater the incidence – which agrees with the SCOPE study.^(23)^

Among the illicit drugs, the only one related to the birth of SGA, in the present study, was *crack*, which is corroborated by data by Mello.^(26)^ Marijuana and cocaine showed no significant relation, similar to the study conducted by Fergusson et al.,^(24)^ and contrary to the review study conducted by Cembranelli et al.^(25)^

The health status of the mother has direct relevance to the fetus.^(1)^ According to Chappell et al.,^(27)^ the frequency of SGA is higher among women with preeclampsia than among those with CH. Similarly, in this study, HDP was associated with the birth of SGA infants.

Odibo et al.,^(17)^ showed that CH is an important risk factor for the birth of SGA babies, but could not find the reason why. However, in this study, there was no relation between CH and the birth of SGA babies.

The birth of SGA infants is a frequent finding in pregnant women with complications of pre-gestational and gestational diabetes.^(1)^ In this study, GDM and pregestational diabetes had no significant statistical association with the birth of SGA babies.

Infections may potentially lead to infectious and teratogenic complications in the fetus.^(1)^ In this study, maternal syphilis was associated with an increased probability of the child being SGA (2.61-fold higher). However, the same was not found for other infections, probably because they have not been extensively investigated during pregnancy.

Oliveira et al.,^(28)^ in a maternity hospital in the city of Juiz de Fora (state of Minas Gerais), found an association between placental infarction, the deposition of intervillous fibrin and lower weight placentas with the birth of SGA babies. In this study, we found that infarction and low placental weight were statistically associated with the birth of SGA babies.

## CONCLUSION

This study found a high frequency of small for gestational age newborns. Many of the causes thereof are widely known and could have been identified, handled, and avoided during prenatal care, if it had been appropriate. The identification of small-for-gestational-age babies in the neonatal period allows an early and appropriate follow-up by a multidisciplinary team, avoiding the consequences and deleterious sequelae.
